# Quality of antibiotic prescribing for pediatric community-acquired Pneumonia in outpatient care

**DOI:** 10.1186/s12887-023-04355-w

**Published:** 2023-10-28

**Authors:** Ariana Saatchi, Manon R. Haverkate, Jennifer N. Reid, Salimah Z. Shariff, Marcus Povitz, David M. Patrick, Michael Silverman, Andrew M. Morris, James McCormack, Fawziah Marra

**Affiliations:** 1 Faculty of Pharmaceutical Sciences, University of British Columbia, 2405 Wesbrook Mall, Vancouver, BC V6T 1Z3 Canada; 2 London Health Sciences Centre, ICES Western, Lawson Health Research Institute, London, ON Canada; 3 Department of Medicine, University of Calgary, Calgary, AB Canada; 4 British Columbia Centre for Disease Control, Vancouver, BC Canada; 5 School of Population and Public Health, University of British Columbia, Vancouver, BC Canada; 6 Faculty of Medicine, University of Western Ontario, London, ON Canada; 7 Sinai Health System, University Health Network and University of Toronto, Toronto, ON Canada

**Keywords:** Antimicrobials, Community acquired Pneumonia, Outpatient care, Pediatrics, Stewardship, Appropriate prescribing

## Abstract

**Background:**

Antibiotics remain the primary treatment for community acquired pneumonia (CAP), however rising rates of antimicrobial resistance may jeopardize their future efficacy. With higher rates of disease reported in the youngest populations, effective treatment courses for pediatric pneumonia are of paramount importance. This study is the first to examine the quality of pediatric antibiotic use by agent, dose and duration.

**Methods:**

A retrospective cohort study included all outpatient/primary care physician visits for pediatric CAP (aged < 19 years) between January 1 2014 to December 31 2018. Relevant practice guidelines were identified, and treatment recommendations extracted. Amoxicillin was the primary first-line agent for pediatric CAP. Categories of prescribing included: guideline adherent, effective but unnecessary (excess dose and/or duration), under treatment (insufficient dose and/or duration), and not recommended. Proportions of attributable-antibiotic use were examined by prescribing category, and then stratified by age and sex.

**Result(s):**

A total of 42,452 episodes of pediatric CAP were identified. Of those, 31,347 (76%) resulted in an antibiotic prescription. Amoxicillin accounted for 51% of all prescriptions. Overall, 27% of prescribing was fully guideline adherent, 19% effective but unnecessary, 10% under treatment, and 44% not recommended by agent. Excessive duration was the hallmark of effective but unnecessary prescribing (97%) Macrolides accounted for the majority on non-first line agent use, with only 32% of not recommended prescribing preceded by a previous course of antibiotics.

**Conclusion(s):**

This study is the first in Canada to examine prescribing quality for pediatric CAP by agent, dose and duration. Utilizing first-line agents, and shorter-course treatments are targets for stewardship.

**Supplementary Information:**

The online version contains supplementary material available at 10.1186/s12887-023-04355-w.

## Introduction

Pneumonia remains one of the leading global causes of mortality particularly in children, and older adults [1, 2]. In Canada, community-acquired pneumonia (CAP) is associated to substantial morbidity, mortality and economic burden [3]. Community-acquired pneumonia (CAP) encompasses infections outside of a hospital setting. Up to 80% of patients with pneumonia are treated in the community, however the etiology of CAP is heterogeneous with both bacterial and viral etiologies prevalent in children [4, 5]. Underlying pathogens can include *S. pneumoniae* however viral infections, and mixed bacterial-viral infections are common, contributing to the complexity of pathogen detection and treatment [6]. In light of this heterogeneity, physicians rarely have knowledge of precise etiology, and treatment is commonly empiric in nature.

The evaluation of outpatient prescribing is often contingent on patient factors not captured within routine, administrative health data. Prior efforts to overcome gaps include the establishment of expected, or maximal, prescribing rates in order to create a benchmark against which antibiotic use may be contextualized [7]. Most often generated using expert consensus methodology, these markers are inherently limited by participant self-selection, physician biases to specific populations, and issues with reliability/external validity. Moreover, these results provide high-level interpretations of *appropriate* use, which has been subject to criticism given the lack of robust patient information to sufficiently elucidate prescription quality. Efforts are further limited by the absence of standardized nomenclature [8].

This retrospective cohort study is the first in Canada to examine the appropriateness of antibiotic prescriptions issued for pediatric community-acquired pneumonia, in outpatient care. In British Columbia (BC), Canada Bugs & Drugs™ is a provincially utilized clinical reference, published in 2012 [9]. The objective of this study was to utilize this resource, as well as other relevant practice guidelines available during the study period, to examine appropriate antibiotic use. The use of guidelines to define categories of prescribing offer a novel, objective interpretation of prescribing quality when compared to previous efforts of expert opinion. Our objectives were to determine the quality of antibiotic prescribing for pediatric CAP by agent, dose and duration. Appropriate use by agent was hypothesized to account for a majority of antibiotic use, while duration of therapy was expected to exceed guideline recommendations.

## Methods

### Data sources

Canadian citizens and permanent residents are covered under provincial health insurance (the Medical Service Plan, i.e. MSP) [10]. The BC Ministry of Health houses health care related databases, which have comprehensive information on BC residents (population: 5 million) [11]. MSP contains all records for services covered by BC’s universal insurance program. Physicians submit claims for services provided, including diagnostic codes. Antibiotic information is logged within BC PharmaNet, a real-time system recording all prescriptions dispensed in the community, as well as hospital outpatient pharmacies [12]. All antimicrobials are recorded except antiretrovirals, as well as medications administered within hospitals and/or emergency departments. The Discharge Abstracts database contains information for provincial acute care hospitals [13]. Patient demographics were provided through a consolidation file [14].

### Study Population

All BC residents with an MSP physician (family practice and pediatricians) record for CAP, from January 1, 2014 to December 31, 2018, were included. Patients aged ≥ 19 years and individuals living in long-term care facilities were excluded. Records were identified using relevant ICD-9 diagnostic codes for bacterial pneumonia (481–486). Diagnoses of viral pneumonia (ICD-9: 480) were not included in the characterization of CAP. A 3-tier diagnostic hierarchy was utilized to identify cases of CAP and account for concurrent diagnoses, this methodology was adapted from Fleming Dutra et al. (2016), and has been previously utilized in Canada [15, 16]. Acute episodes of infection were defined using a 14-day window. All subsequent physician visits within 14 days were flagged as a single episode with the first as the index date. Chronic episodes exceeding 30 days were excluded. Any patients admitted to hospital within 5 days of an episode were also excluded, as medications dispensed in hospital are not available through PharmaNet. Moreover, these patients are likely to have received inpatient treatment. These criteria have previously been used to identify episodes of infection in Canadian outpatient care [17].

Antibiotic dispensations were extracted from PharmaNet and matched to MSP using anonymized patient identifiers. A prescription was linked using an algorithm that matched the date on which the medication was dispensed to a permissible time-frame. This range included from index date to episode end, with an additional 5-day follow up. If multiple prescriptions were present within a period, only the first was kept to examine empiric prescribing only. Antibiotics were classified based on the Anatomical Therapeutic Chemical (ATC) classification system developed by the WHO [18]. Multiple episodes of infection, and subsequent prescriptions per subject were permitted in our analyses. All raw data with n < 6 were excluded from analyses to preserve subject anonymity.

### Categories of prescribing quality

Study categories of prescribing quality included: (1) guideline adherent;(2)under treatment;(3) effective but unnecessary; and(4) not recommended. Guideline adherent, effective but unnecessary and under treatment encompassed first-line agent use, and were delineated by dose and duration parameters. Not recommended included all non-first-line agent prescriptions. Supplementary Table 1 summarizes inclusion criteria for each category of prescribing quality. These categories were adapted from Dresser et al. (2017) [19].

### Clinical guidelines, dose and duration

Clinical practice guidelines and treatment recommendations were extracted from Bugs & Drugs, a provincial reference, as well as: American Academy of Pediatrics (AAPS), Pediatric Infectious Diseases Society (PIDS), Infectious Disease Society of America (IDSA), Canadian Pediatric Society (CPS), and the British Thoracic Society (BTS) [4, 6, 9, 20, 21]. Guidelines were reviewed and first-line agent(s), dosing and duration extracted. Treatment recommendations for β-lactam allergies were excluded as medication allergies are recorded but remain unavailable in PharmaNet data.

Amoxicillin [40-90 mg/Kg/day; max 4 g/day; x 5–7 days] was the primary treatment for outpatient pediatric CAP. Alternative first-line treatments included: Penicillin [25-50 mg/Kg/day; max 2 g/day; x 5–7 days]; Ampicillin [50-100 mg/Kg/day; max 2 g/day; x 5–7 days]; and Amoxicillin-clavulanate [90 mg/Kg/day; max 4 g/Kg/day; x 5–7 days]. Average daily dose was stratified by patient age, and calculated utilizing WHO growth charts for Canada [22]. For each age, a daily dosing range was calculated. The 3rd and 97th percentile weights at the mid-point (i.e. 6.5 years for a 6-year-old) were determined for each sex. Per age and sex, the lowest 3rd percentile weight was multiplied by the lowest dose (e.g. 40 mg for amoxicillin) to calculate the minimum daily dose. The highest 97th percentile was multiplied by the highest dose (e.g. 90 mg for amoxicillin) to calculate the maximum; up to the maximum daily dose stated in guidelines (e.g. 4 g for amoxicillin).

Published in 2012, provincial guidelines state that 5–7 days is the appropriate duration of antibiotic treatment [9]. Other guidelines put forth by the AAPS and IDSA, during our study period, do include longer durations (7–10 days), however given the scope of our study is limited to BC, regionally-specific recommendations were prioritized [21].

### Outcomes & sensitivity analyses

Baseline characteristics were examined by age, sex, income quintile and rurality. Primary outcomes included: the proportion of total CAP episodes prescribed; the proportion of antibiotic use by category; and changes in trends over time. Categories were further examined to identify proportions attributable to deviations in dose and/or duration. Primary outcomes included amoxicillin as a first-line agent. Secondary outcomes also included penicillin, ampicillin, and amoxicillin-clavulanate, alongside amoxicillin. Results were then stratified by age and sex.

A sensitivity analysis was determined *a priori* to examine weight-based dosing. As average daily dose was calculated using growth charts, a secondary analysis was completed using the 25th and 75th markers to narrow the range for guideline adherence and examine more conservative dosing.

Two post hoc analyses were also completed. The first examined the prevalence of prior antibiotic utilization for non-first line prescriptions dispensed for CAP, and categorized as *Not Recommended*. Using a 90-day lookback window, prescriptions were reviewed overall, and by drug class. Given the impact of prior β-lactam use upon patient susceptibility—the majority of “not recommended”  use was hypothesized to have a preceding antibiotic. Second, we examined appropriate use in two pediatric subgroups: (1) ≤ 5 years; and (2) 12–18 years, to compare prescribing quality in the context of varying prevalence of *Mycoplasma pneumoniae*.

## Results

Our study period included a total of 28,307 patients, with 54% male and a mean age of 6.5 years (Table 1). Overall, there were 42,452 episodes of pediatric CAP in BC, between 2014 and 2018. Of those, 32,469 episodes resulted in a prescription, with a proportion of 76% total CAP episodes prescribed (Table 1). The age distribution of patients by sex shows that 72% of antibiotics were dispensed to children aged 1–6 years (Supplementary Fig. 1).

**Table 1:** Cohort Characteristics

		2014	2015	2016	2017	2018	Overall	
N		6351 (22.5%)	6130 (21.7%)	6047 (21.4%)	5120 (18.2%)	4659 (16.5%)	28,307	
Age	Mean (SD)	6.64 (4.81)	6.93 (4.87)	6.54 (4.91)	6.57 (5.07)	5.86 (4.77)	6.54 (4.90)	
	Median (IQR)	5 (3–10)	6 (3–10)	5 (2–10)	5 (2–10)	4 (2–8)	5 (2–10)	
	0–2 years	1471 (23.2%)	1320 (21.5%)	1559 (25.8%)	1397 (27.3%)	1460 (31.3%)	7207 (25.5%)	
	3–9 years	3249 (51.2%)	3087 (50.4%)	2956 (48.9%)	2420 (47.3%)	2232 (47.9%)	13,944 (49.3%)	
	10–18 years	1631 (25.7%)	1723 (28.1%)	1532 (25.3%)	1303 (25.4%)	967(20.8%)	7156 (25.3%)	
Sex	F	2896 (45.6%)	2840 (46.3%)	2812 (46.5%)	2370 (46.3%)	2188 (47%)	13,054 (46.3%)	
	M	3455 (54.4%)	3290 (53.7%)	3235 (53.5%)	2750 (53.7%)	2471 (53%)	15,144 (53.7%)	
Income Quintile^1^	Missing	110 (1.7%)	93 (1.5%)	77 (1.3%)	58 (1.1%)	76 (1.6%)	413 (1.5%)	
	1	1239 (19.5%)	1180 (19.2%)	1074 (17.8%)	959 (18.7%)	860 (18.5%)	5298 (18.8%)	
	2	1301 (20.5%)	1319 (21.5%)	1207 (20%)	996 (19.5%)	912 (19.6%)	5723 (20.3%)	
	3	1299 (20.5%)	1187 (19.4%)	1232 (20.4%)	1061 (20.7%)	943 (20.2%)	5715 (20.3%)	
	4	1239 (19.5%)	1205 (19.7%)	1324 (21.9%)	1102 (21.5%)	987 (21.2%)	5847 (20.7%)	
	5	1163 (18.3%)	1146 (18.7%)	1133 (18.7%)	944 (18.4%)	881 (18.9%)	5252 (18.6%)	
Rural^2^	Missing	247 (3.9%)	185 (3%)	191 (3.2%)	123 (2.4%)	134 (2.9%)	880 (3.1%)	
	N	1063 (16.7%)	1056 (17.2%)	1077 (17.8%)	871 (17%)	807 (17.3%)	22,510 (79.8%)	
	Y	5041 (79.4%)	4889 (79.8%)	4779 (79%)	4126 (80.6%)	3718 (79.8%)	4811 (17.1%)	
Total Cap Episodes^3^ (N)		9768	9151	8872	7776	6885	42,452	
Prescribed Episodes^4^ (N)		7138	7166	6929	5977	5259	32,469	
Proportion Prescribed^5^		0.73	0.78	0.78	0.77	0.76	0.76	

*1 Population Data BC determines neighborhood income quintile (i.e. household size-adjusted measure of household income) using a postal code-based algorithm standardized by Statistics Canada; 2 Rural status represents local population of 1000 to 29 999; urban status represents local population ≥ 30 000; 3 acute episodes (< 30 days) of pediatric CAP regardless of associated-antibiotic prescription; 4 acute episodes (< 30 days) that received an antibiotic prescription within 5 days of episode end date; 5 proportion prescribed as: [# episodes prescribed / # total episodes]*

**Fig. 1:**
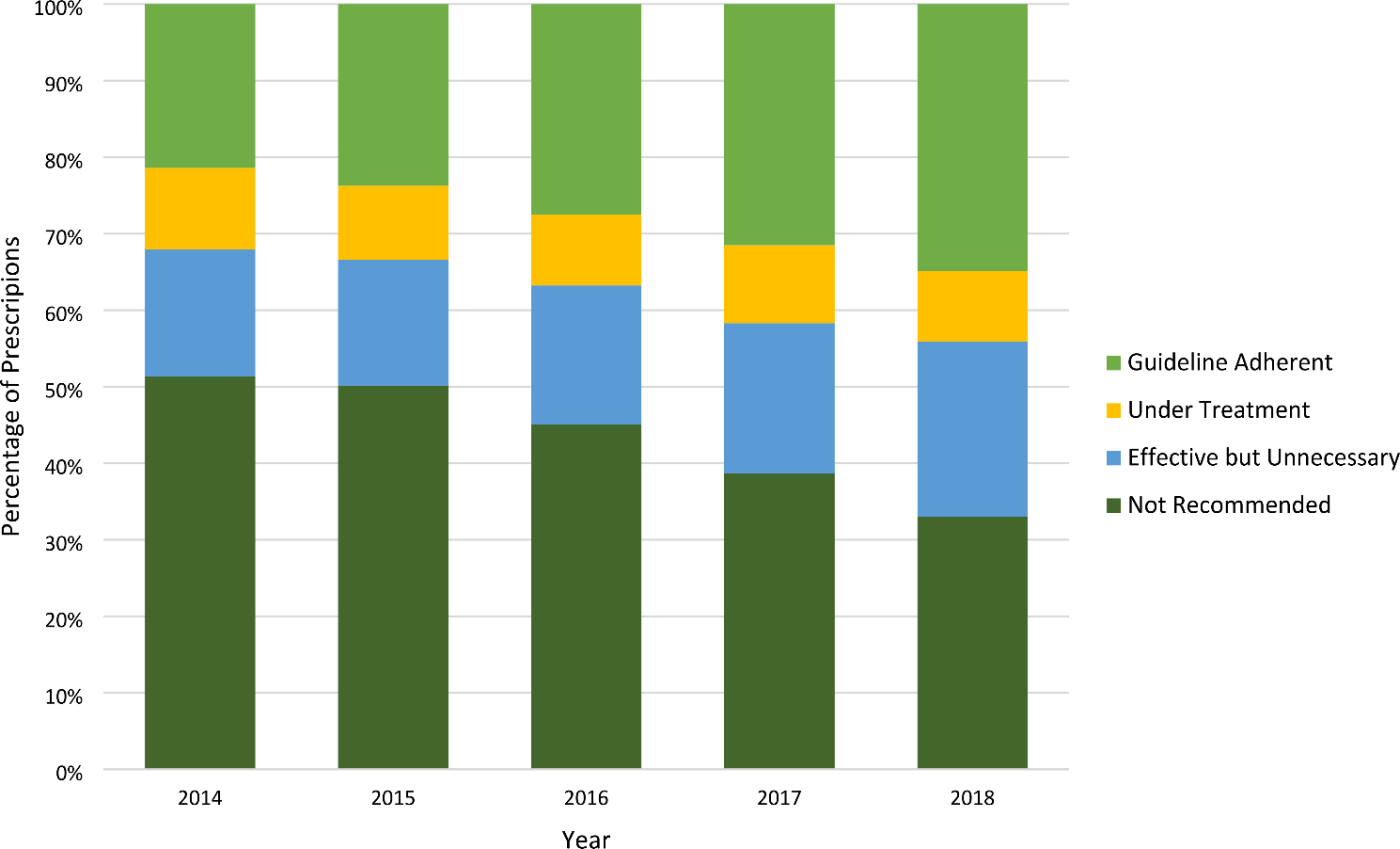
Percentage of outpatient antibiotic use for pediatric CAP by category of prescription quality by year

Amoxicillin comprised 51% of all prescriptions in the five study years. The majority (53%) of amoxicillin prescriptions were categorized as fully guideline adherent. Alternative first-line treatments for CAP included: amoxicillin-clavulanate, ampicillin and penicillin, which accounted for an additional 5% of prescribing, for a total of 56% first-line agent use. Overall, 44% of prescriptions were classified as not recommended, followed by 27% guideline adherent, 19% effective but unnecessary, and 10% under treatment (Fig. 1).

When compared to 2014, guideline adherent prescribing significantly increased by the final study year [RR: 1.7; 95% CI: 1.6–1.8] (Table 2). Effective but unnecessary use also demonstrated an increase, while under treatment and not recommended prescribing decreased (Table 2). Durations exceeding 5–7 days were the primary characteristic of inappropriate prescribing, accounting for 97% of all effective but unnecessary antibiotic use (Supplementary Table 2). Sub therapeutic dosing was responsible for 50% of under treatment antibiotic use, while the remainder could be attributed to both sub therapeutic dosing *and* excess duration.

**Table 2:** Rate of antibiotic use for pediatric CAP by category of prescribing quality

Prescribing Category	Rate of Prescribing per 1000 Population					Rate Ratios^1^ (95% CI)
	2014	2015	2016	2017	2018	
Guideline Adherent^2^	249	287	329	385	418	1.68 (1.57–1.80)
Effective but Unnecessary^3^	194	199	217	240	275	1.42 (1.31–1.54)
Under Treatment^4^	124	117	111	124	111	0.89 (0.80–1.00)
Not Recommended^5^	596	605	538	472	396	0.66 (0.63–0.70)

*1 rate ratios compare prescribing in 2018 against 2014; 2 guideline adherent: concordant to first-line agent, dose and duration; 3 effective but unnecessary: concordant to first-line agent with dose and/or duration discordant; 4 under treatment: concordant to first-line agent with dose and/or duration discordant; 5 not recommended: discordant to first-line agent*

**Fig. 2:**
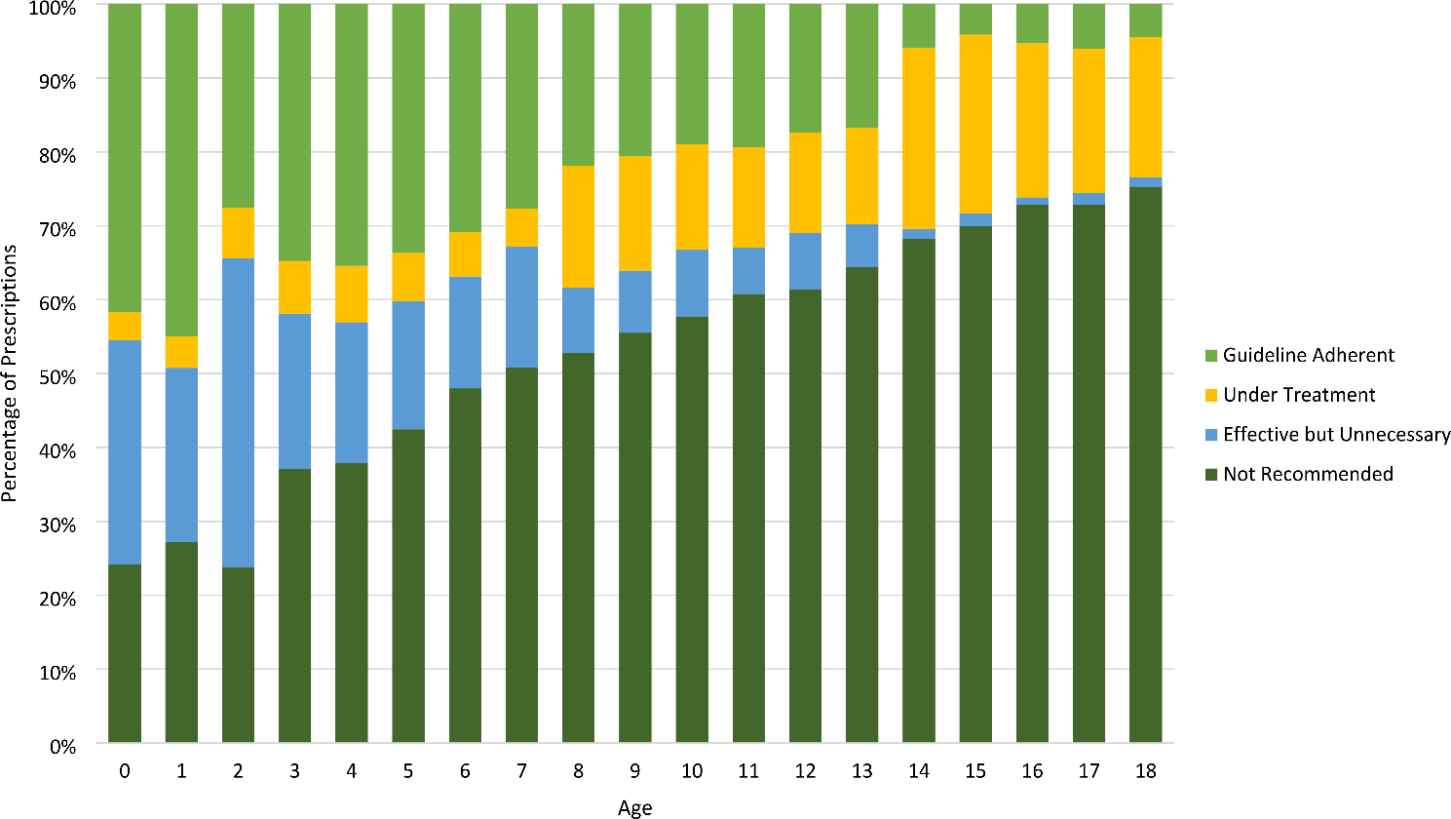
Overall percentage of outpatient antibiotic use for pediatric CAP by category of prescription quality by age

Stratified by age, patterns of use vary across prescribing categories (Fig. 2). Guideline adherent prescriptions were most pronounced in children aged < 2 years. Effective but unnecessary use was highest in the youngest ages, with a negative association to increasing age. By age 14, only 1% of prescriptions were effective but unnecessary, in contrast to 25% in children aged ≤ 1 year. Under treatment demonstrated an opposite shift over time, with increasing age leading to increased proportions, particularly in children aged 14 years and older wherein under treatment accounted for 21% of antibiotic use, compared to 4% in younger ages. Not recommended prescriptions accounted for the highest magnitudes of use for children aged ≥ 3 years, with a positive association to increased age. By age 18, 75% of antibiotic use was categorized as not recommended. In contrast, guideline adherent accounted for the majority of prescriptions issued to children aged ≤ 2 years (35%). When stratified by sex, no marked differences were revealed.

### Not recommended prescribing

In total, 56% of antibiotics were concordant with guideline recommended first-line treatments. The remaining 44% were composed of other antibiotic agents, and subsequently categorized as “not recommended”. By the end of our study period, proportions of not recommended prescribing decreased by 44%, suggesting a positive trend towards guideline concordant first-line use [RR: 0.66: 95% CI: 0.6–0.7].

The majority of “not recommended” (86%) antibiotics were macrolides (ATC class: J01F). Moreover, 73% of these prescriptions could be attributed to either clarithromycin (44%), or azithromycin (29%) specifically. Other antibiotics included those belonging to ATC class J01D (other β-lactams; 9%); and J01A (tetracyclines; 3%). The remaining 1.7% of “not recommended” antibiotics were other agents not listed above.

### Sensitivity & Post Hoc analyses

The sensitivity analysis on weight-based dosing showed minor changes when compared to the main analysis. Small decreases could be seen in the proportions of “guideline adherent” and “effective but unnecessary” prescribing, while the proportion of undertreatment increased. Overall, changing these parameters did not have a significant impact on trends of appropriateness. Supplementary Fig. 2 provides age-specific plots for frequency of dosing, using the 25th and 75th weight percentiles.

An examination of duration frequencies revealed 60% of all amoxicillin prescriptions were 7-days in duration, followed by 10 days (31%), and 5 days (6%). (Supplementary Fig. 3). Overall, 67% of durations dispensed were fully guideline adherent (5–7 days). A subgroup analysis by age showed similar trends in use across categories of quality as previously discussed (Supplementary Fig. 4).

Post hoc analyses revealed that 68% of not recommended antibiotic use did not have a preceding PharmaNet antibiotic record. For the 32% with a prior dispensation, on average, 54% of non-first line antibiotic use was observed to have had a preceding β-lactam prescription. For macrolide use specifically, 70% were preceded by a β-lactam. Supplementary Table 3 contains further information by drug class.

## Discussion

Our study showed that first-line agents, concordant with guideline recommendations, accounted for over half of prescriptions issued for pediatric CAP while a remaining 44% of prescriptions were categorized as “not recommended”. Overall, 27% of antibiotic use in BC was fully guideline adherent. Prescriptions classified as effective but unnecessary, were 97% attributable to excessive duration of therapy. Moreover, half of under treatment was attributable to subtherapeutic dose in tandem to excess duration. Provincial pediatric guidelines have included evidence for shorter durations since 2012, preceding our study period [9]. IDSA/PIDS 2011 guidelines also cite *strong recommendations* for shorter duration despite *moderate* evidence at the time [21]. Results for several clinical trials have since been disseminated which provide concrete evidence in support of short course treatment [23–25].

To our knowledge, this is the first study examining agent, dose and duration, to assess antibiotic prescribing in pediatric CAP. Although Frost et al. evaluated guideline-concordant prescribing for upper respiratory illnesses in children, they did not consider dose or duration [26]. Furthermore, a study in China assessed prescribing for respiratory infections and found that the three most prescribed agents were discordant with guideline recommendations, but dose/duration was not considered [27].

The results of our study indicate that the quality of antibiotic prescribing for pediatric CAP could be improved in numerous ways. Agent selection and duration of therapy most significantly, as these two components of prescribing account for 86% of total inappropriate antibiotic use. Further analyses revealed that the use of macrolides (e.g. azithromycin and clarithromycin) accounted for almost all non first-line agent prescriptions. Macrolides are *only* indicated if the patient has a severe β-lactam allergy, or if an atypical pathogen (e.g. *Mycoplasma* or *Chlamydia* species) is suspected as the causative pathogen [4, 9, 21]. Although both allergy and etiology data were unavailable, it is improbable that 44% of all BC children met these criteria. Moreover, provincial data on bacterial resistance and susceptibility patterns do not support the empirical replacement of β-lactams with macrolides, given the resistance rates to *S.pneumoniae* are close to 25%, the cut-off suggested by IDSA guidelines to avoid empiric macrolide use [28, 29]. A previous study reviewing antibiotic use in Canada also found high macrolide use, and this has been corroborated across several studies in the United States (US) [30–33]. Lipsett et al. report that 43% of all antibiotic prescribing for pediatric CAP in the US is attributable to macrolide monotherapy, with a positive association between increasing age and macrolide prescribing [33]. We also showed a clear association between increasing levels of “not recommended” prescribing, with increasing patient age. Although atypical etiology is more common in older children/adolescents, amoxicillin is still recommended, with a macrolide to follow in treatment failure. A post-hoc analysis revealed that only 32% of not recommended prescribing was preceded by a different course of antibiotics. Of these prescriptions, ¾ were macrolides, and 70% were preceded by a β-lactam prescription. This result leaves a significant proportion of not recommended prescribing unexplained by the suspicion of *Mycoplasma pneumoniae* or other atypical pathogens. Although macrolide antibiotics may be justified in the treatment of atypical pathogenesis, *S. pneumoniae* remains the most common pathogen for pediatric CAP [34]. Given that not recommended prescribing accounted for up to ¾ of antibiotic use in older children, it is unlikely that the empiric use of macrolides can be fully explained by true atypical infection. Reducing the empiric use of broad-spectrum macrolides in the wake of rising Canadian rates of drug resistant *M. pneumoniae* presents a target for provincial stewardship programs [35].

Appropriate duration of therapy was identified as 5–7 days, based on clinical guidelines available during the study period. More recently, the SAFER, SCOUT-CAP, and CAP-IT trial results have become available, which offer robust clinical evidence in favour of short course treatments (3–5 days) [23–25]. These randomized, multicenter, trials enrolled children (N = 1485) with non-severe CAP. The SAFER trial was a blinded non-inferiority study completed in Canadian emergency departments, concluding that 5-day high-dose amoxicillin therapy was comparable to 10-day. In the United States, the SCOUT-CAP trial examined outpatient, urgent care, as well as emergency settings, and corroborate SAFER results. Moreover, SCOUT-CAP offered the first evidence of reduced antibiotic selective pressure resulting from shorter-courses, by examining antibiotic resistance genes (ARGs) in a post-hoc microbiome analysis. The trial concluded that 5-day treatment reported significantly lower levels of ARGs when compared to longer durations. The CAP-IT non-inferiority trial concluded that 3-day treatment was non-inferior to 7 days of amoxicillin. With results published in 2021/22, all three trials provide irrefutable clinical evidence that short-course should be standard-of-care for non-severe pediatric CAP infection. Across multiple outcomes of interest including: treatment failure, hospitalization, adverse events, short-course (3–5 days) treatments have been identified as equally safe and effective when compared to longer durations (7–10 days) [36]. Moreover, shorter durations of therapy increased year-on-year, while 10-day dispensations decreased, indicating an existing trend towards short-course treatment within BC prior to trial result dissemination. The Do Bugs Need Drugs stewardship campaign has been operating at the provincial-level within BC since 2005 [37]. These study results offer discrete targets for intervention:(1) encouraging the use of β-lactam antibiotics in lieu of empiric macrolide use, and (2) continued emphasis on 5–7 days of therapy to reduce the risk of bacterial resistance.

This study has limitations inherent to all retrospective studies using administrative health data. Data utilized are limited to 2014 through 2018, however reported results remain informative given the population-level data for millions of Canadian children on medications, physician visits, and hospitalizations. Although routinely utilized for research, the original purpose of these indices is insurance billing, and as such there is a delay between data capture and availability for research engagement. Moreover, many researchers utilizing these data are excluding years impacted by the SARS-CoV-2 pandemic as variable antibiotic use during this time period has been identified [38, 39].Further research efforts are underway to characterize more recent data upon the availability of subsequent years. Case identification is reliant on accurate coding by physicians. We identified that 24% of pediatric CAP cases were not prescribed an antibiotic—despite receiving a diagnosis that would warrant their utilization—this may be partially explained by the current promotion of delayed-antibiotic prescribing as encouraged by provincial stewardship efforts. Further, as lab data did not confirm the presence of infection, use of ICD-9 codes may be subject to misclassification bias. Canadian physician claims data have high positive-predictive value for respiratory infections, however studies on validity specific to BC administrative health data is lacking [40]. Similarly, as culture results were not available to identify atypical infection, our interpretations of inappropriate macrolide use is limited to empiric prescribing, and preceding antimicrobial use. As unfilled prescriptions are not included within dispensation records, our discussion of antibiotic prescribing may be an underrepresentation of provincial use. Relatedly, adherence to medications dispensed is unknown. Only empiric prescriptions were examined and study results cannot be extrapolated to treatment failures or secondary treatment courses. Dose and duration were reported based on dispensation records, patient adherence and/or physician instructions were not available. Moreover, dose was not available as a discrete variable rather average daily dose was calculated as follows: [(drug strength*quantity dispensed)/days of therapy]. Utilizing WHO Canadian growth charts in the absence of true patient weight is also a limitation, however in 2017 only 10% of BC children were reported obese. First-line treatments were extracted from clinical guidelines/resources, with amoxicillin flagged as primary treatment based on regional recommendations. The delineation of primary/alternative treatments is not a reflection on spectrums of activity. Our characterization of prescribing quality is limited by the absence of additional patient factors not captured within routine administrative health data. Patient presentation, symptom severity, and allergy data were not recorded within the utilized indices and as such, our characterization of prescribing quality is limited to diagnostic code. Allergy data was not available in our administrative datasets as this is a text field recorded in the pharmacy database that is not readily searchable. The absence of allergy data limited our characterization of non-first-line antibiotic use to “not recommended”. The future availability of accurate β-lactam allergy records may provide more nuance, and the inclusion of a clinical justification for otherwise inappropriate prescriptions. Future research efforts should also engage with laboratory data to identify cases of *atypical* infection to further delineate macrolide use.

## Conclusion

Continued surveillance of antibiotic use is integral to dissuade bacterial resistance. Despite decades of surveillance evaluating quantity, this is the first study to examine outpatient antibiotic prescribing quality for pediatric CAP. Moreover, this study is the first in Canada to examine prescribing quality by agent, dose and duration. Promoting the use of first-line agents, and shifting to reflect evidence-based short-course treatment are targets for stewardship. Study results highlight the importance of examining the quality of prescriptions across other common indications, to inform and equip stewardship interventions.

### Electronic supplementary material

Below is the link to the electronic supplementary material.

Supplementary Material 1

## Data Availability

The data that support the findings of this study are available from Population Data BC, but restrictions apply to the availability of these data, which were used under license for the current study and so are not publicly available. The data are, however, available from the authors upon reasonable request and with the permission of Population Data BC. For data inquiries please contact Dr. Fawziah Lalji (Marra) (fawziah@mail.ubc.ca).

## Abbreviations

CAP: community acquired pneumonia
BC: British Columbia
MSP: Medical Services Plan
ICD: international statistical classification of diseases and related health problems
WHO: world health organization
ATC: anatomical therapeutic chemical
AAPS: American Academy of Pediatrics
PIDS: Pediatric Infectious Diseases Society
IDSA: Infectious Disease Society of America
CPS: Canadian Pediatric Society
BTS: the British Thoracic Society
DOT: duration of therapy
ARGs: antibiotic resistance genes
