# Supplementary material for: Quality of antibiotic prescribing for pediatric community-acquired Pneumonia in outpatient care
Source: BMC Pediatr. 2023 Oct 28;23:542. doi: 10.1186/s12887-023-04355-w (PMC10612244; doi:10.1186/s12887-023-04355-w)
Supplement: Supplementary file 1 — Supplementary Material 1 [file 12887_2023_4355_MOESM1_ESM.docx]

Supplementary Figure 1. Population pyramid of antibiotic use (%) for pediatric CAP, by age and sex

Supplementary Figure 2. Number of prescriptions issued by average daily dose, per patient age, with 25^th^ and 75^th^ percentile markers

Supplementary Figure 3. Distribution of amoxicillin prescribing by duration of therapy

Supplementary Figure 4. Overall percentage of outpatient antibiotic use for pediatric CAP by category of prescription quality by age category

Supplementary Table 1. Categories of prescribing quality

| **Prescription Quality** | **Definition** |
| --- | --- |
| **Guideline Adherent** | Guideline concordant first line agent, dose, duration |
| **Under Treatment** | Guideline concordant first line agent that can be improved in one of the following categories: (1) drug dose (*low* in comparison to guideline recommendation); (2) duration of therapy (*short* in comparison to guideline recommendation)   1. Dose low; Duration guideline adherent 2. Dose guideline adherent; Duration short 3. Dose low; Duration short 4. Dose low; Duration long 5. Dose high; Duration short |
| **Effective but Unnecessary** | Guideline concordant first line agent that can be improved in one of the following categories: (1) drug dose (*high* in comparison to guideline recommendation); (2) duration of therapy (*long* in comparison to guideline recommendation)   1. Dose high; Duration guideline adherent 2. Dose guideline adherent; Duration long 3. Dose high; Duration long |
| **Not Recommended** | Guideline discordant non-first-line agent |

| Age (years) | **Effective but Unnecessary** | | |  | |
| --- | --- | --- | --- | --- | --- |
|  | EXCESS DOSE^1^ ADHERENT DURATION^2^ | ADHERENT DOSE  LONG DURATION | EXCESS DOSE  LONG DURATION |  |  |
| 0 - 5 | 6.50 | 92.29 | 1.21 |  |  |
| 6 - 11 | 2.07 | 97.93 | 0.00 |  |  |
| 12 - 18 | 0.00 | 100.00 | 0.00 |  |  |
|  | **Under Treatment** | | | | |
|  | LOW DOSE  ADHERENT DURATION | ADHERENT DOSE SHORT DURATION | LOW DOSE  SHORT DURATION | EXCESS DOSE  SHORT DURATION | LOW DOSE LONG DURATION |
| 0 - 5 | 36.87 | 14.67 | 0.00 | 7.92 | 40.54 |
| 6 - 11 | 45.07 | 10.43 | 0.00 | 0.00 | 44.51 |
| 12 - 18 | 58.79 | 1.54 | 1.28 | 0.00 | 38.38 |
| *1 Dose was calculated using WHO Canadian Growth charts, 3^rd^ and 97^th^ percentiles were utilized to calculate adherent upper and lower bounds for average daily dose per pediatric age; 2 Duration of 5 – 7 days were considered adherent, with < 5 and > 7 utilized as markers for short and long duration of therapy, respectively* | | | | | |

Supplementary Table 2. Relative proportion of discordant dose and/or duration first-line agent use, by age.

Supplementary Table 3. Relative proportion of antibiotic dispensed ≤ 90 days prior to “Not Recommended” prescription for pediatric CAP, by major anatomical therapeutic class

|  | Antibiotic class dispensed ≤ 90 days prior | | | | | |
| --- | --- | --- | --- | --- | --- | --- |
| Antibiotic class dispensed for CAP | Tetracyclines | β-lactam Penicillin | Other β-lactams | Macrolides | Quinolones | Other Antibacterials^1^ |
| Tetracyclines | 11.4% | 46.3% | 6.5% | 27.6% | 1.6% | 6.5% |
| β-lactam Penicillin | 0.9% | 65.6% | 8.8% | 20.0% | 0.4% | 4.2% |
| Other β-lactams | 0.3% | 58.6% | 12.4% | 24.6% | 0.3% | 3.7% |
| Macrolides | 1.1% | 70.3% | 7.6% | 18.0% | 0.4% | 2.6% |
| Quinolones | 3.8% | 43.0% | 3.8% | 38.0% | 8.9% | 2.5% |
| Other Antibacterials^1^ | 1.9% | 38.9% | 5.6% | 16.7% | 0.0% | 37.0% |
| *1 Other Antibacterials describe ATC class J01X antibiotics including: nitrofurantoin, metronidazole, fosfomycin and more* | | | | | | |
